# Predictors of Severe Herpes Zoster: Contributions of Immunosenescence, Metabolic Risk, and Lifestyle Behaviors

**DOI:** 10.3390/diseases14010026

**Published:** 2026-01-08

**Authors:** Mariana Lupoae, Fănică Bălănescu, Caterina Nela Dumitru, Aurel Nechita, Mădălina Nicoleta Matei, Simona Claudia Ștefan, Alin Laurențiu Tatu, Elena Niculet, Alina Oana Dumitru, Andreea Lupoae, Dana Tutunaru

**Affiliations:** 1 Faculty of Medicine and Pharmacy, “Dunarea de Jos” University of Galati, 800008 Galați, Romania; 2 Centre in the Medical-Pharmaceutical Field, Faculty of Medicine and Pharmacy, “Dunarea de Jos” University of Galati, 800008 Galati, Romania; 3 Emergency Clinical Hospital “Sf. Apostol Andrei”, 800010 Galati, Romania

**Keywords:** herpes zoster, varicella-zoster virus, immunosenescence, postherpetic neuralgia, ophthalmic zoster, lifestyle factors, metabolic dysfunction, epidemiology, risk factors, Romania

## Abstract

Background: Herpes zoster (HZ) represents a substantial public health concern among aging populations, yet regional variability in clinical patterns and risk determinants remains insufficiently documented. In southeastern Romania, epidemiological data are limited, and the combined influence of demographic, behavioral, and metabolic factors on disease severity has not been systematically evaluated. Methods: We performed a retrospective observational study including 100 consecutive patients diagnosed with HZ between 2019 and 2023 in a dermatology department in southeastern Romania. Demographic characteristics, lifestyle behaviors, anthropometric status, clinical manifestations, and outcomes were extracted from medical records. Associations between categorical variables were assessed using Chi-square tests and Cramer’s V, while interaction patterns were explored through log-linear modeling. Heatmaps were generated in Python (version 3.10) using the Matplotlib library (version 3.7.1) to visualize distribution patterns and subgroup relationships. Results: The cohort showed a marked age dependence, with 77% of cases occurring in individuals ≥ 60 years, consistent with immunosenescence-driven reactivation. Women represented 59% of cases, and 84.7% of female patients were postmenopausal. Urban residents predominated (91%). Vesicular eruption (84%) and acute pain (79%) were the most frequent symptoms. Localized HZ was observed in 81% of cases, while ophthalmic involvement (11%) and disseminated forms (8%) were less common. Lifestyle factors significantly influenced clinical severity: smokers, alcohol consumers, and sedentary individuals exhibited higher proportions of postherpetic neuralgia (PHN) and ocular complications (*p* < 0.001). Overweight and obese patients demonstrated a higher burden of PHN, suggesting a role for metabolic inflammation, although BMI was not associated with incidence. No significant association between age category and complication type was detected, likely due to small subgroup sizes despite a clear descriptive trend toward increased severity with advanced age. Conclusions: These findings support a multifactorial model of HZ severity in southeastern Romania, shaped by age, lifestyle behaviors, hormonal status, and metabolic risk. While incidence patterns align with international data, the strong impact of modifiable factors on complication rates highlights the need for targeted prevention and individualized risk assessment. Results offer a regional perspective that may inform future multicenter investigations.

## 1. Introduction

HZ remains a significant public health concern, with a rising global incidence and a substantial clinical impact on older adults and individuals with impaired immune function [1]. Reactivation of the varicella–zoster virus (VZV), which infects more than 90% of the population during childhood, leads to a painful vesicular eruption with dermatomal distribution and may cause considerable functional impairment [2]. In Europe, the incidence ranges from 2 to 4 cases per 1000 person-years in younger adults and increases to 7–10 cases per 1000 person-years in the elderly [3,4], with similar trends reported in North America and the Asia–Pacific region [5,6].

PHN is the most debilitating complication, affecting up to one-third of patients and exerting a quality-of-life burden comparable to that of major chronic diseases [1,7]. Neurological, ocular, and disseminated forms further contribute to disease severity, particularly among older individuals.

Control of VZV latency depends on the integrity of cell-mediated immunity. VZV-specific T lymphocytes inhibit subclinical viral replication [8,9,10], whereas the age-related decline in cellular immunity—characterized by reduced naïve T cells, accumulation of senescent subsets, and diminished IFN-γ secretion—facilitates viral reactivation [11,12]. Structural alterations of sensory ganglia and lymphoid tissues, including ganglionic involution and dendritic cell dysfunction, additionally impair immune memory and increase susceptibility to HZ [13].

International demographic studies consistently show a higher risk among women (RR ≈ 1.31), alongside relatively stable ethno-racial differences across populations [14]. Emerging data from genetics and immunometabolism suggest that indicators of body adiposity may correlate with the risk of HZ, potentially through mechanisms involving metabolic inflammation and accelerated immunosenescence [15]. Geographic and climatic factors may further modulate epidemiology: studies from Taiwan and China have linked variations in temperature, UV exposure, and humidity to changes in HZ incidence, possibly through effects on cell-mediated immune function [16,17].

These findings highlight the multifactorial nature of host vulnerability to HZ, shaped by the interaction of demographic, immunological, metabolic, and environmental determinants. Nevertheless, how these factors manifest within specific regional contexts remains insufficiently explored, particularly in Eastern Europe.

The aim of this study is to characterize the clinical and epidemiological profile of patients with HZ from southeastern Romania, examining demographic and clinical distributions, patterns of complications, and variables associated with disease severity in a region that remains underrepresented in the literature.

## 2. Materials and Methods

### 2.1. Study Design and Setting

This retrospective observational study was conducted in the Dermatology Department of a hospital in southeastern Romania and included all consecutive patients diagnosed with HZ between 2019 and 2023. All patients underwent clinical and paraclinical evaluation and received therapeutic management in accordance with current medical standards. The study relied exclusively on clinical and epidemiological data recorded in medical charts, with no additional diagnostic or therapeutic interventions, in compliance with national regulations on patient rights and medical research ethics.

Given the retrospective and single-center design, the study was conceived primarily as a descriptive and exploratory analysis of demographic, lifestyle, and clinical patterns in an understudied regional population.

### 2.2. Eligibility Criteria

Patients were eligible for inclusion if they had a confirmed clinical diagnosis of HZ, were aged ≥10 years, and had complete medical documentation during hospitalization, providing information on demographic characteristics, behavioral factors, body weight status, physical activity level, and clinical presentation. Both continuously hospitalized patients and those reevaluated after initial admission were included, provided that their medical records were complete and accessible for retrospective analysis.

Exclusion criteria comprised: uncertain diagnosis or cutaneous lesions incompatible with HZ, incomplete medical files, complex dermatological conditions that could not be reliably differentiated at presentation, and cases with unclear nosocomial onset.

Because of the retrospective design and reliance on routinely collected clinical data, specific exclusion criteria related to immunosuppressive conditions were not systematically applied. Information regarding HIV infection, hematologic or solid-organ malignancies, prior chemotherapy or radiotherapy, chronic systemic corticosteroid therapy, biologic or post-transplant immunosuppressive treatments, and autoimmune diseases was not consistently available in medical records and could therefore not be analyzed as independent variables.

### 2.3. Variables and Clinical Definitions

For each patient, demographic data (age, sex, residence), behavioral characteristics (smoking status, alcohol consumption), and body mass index (calculated using the standard formula and categorized according to WHO criteria) were recorded. Physical activity level was classified as sedentary, light, moderate, or vigorous based on information documented in medical records.

Lifestyle-related variables (smoking, alcohol consumption, and physical activity) were extracted from standardized admission anamnesis notes and classified into predefined categorical groups, without quantitative assessment of intensity, duration, or cumulative exposure.

As these variables were derived from routinely documented clinical records, no validated questionnaires or prospective measurements were available, and the potential for recall and reporting bias is acknowledged.

Seasonality was defined according to the season in which disease onset occurred. Clinical presentation was classified according to international guidelines into localized HZ, ophthalmic HZ, disseminated HZ, and disseminated HZ with varicelliform eruption. Clinical evolution was categorized as uncomplicated HZ, PHN, or ocular complications. Additionally, key symptoms were documented, including pain, vesicular rash, fever, erythema, hyperesthesia, asthenia, pruritus, photophobia, edema, and headache.

### 2.4. Statistical Analysis

Data were entered into an electronic database and manually verified for accuracy. Records with missing essential data were excluded without imputation, and analyses were performed on the complete-case dataset. Continuous variables were summarized using mean, median, and standard deviation, whereas categorical variables were expressed as absolute frequencies and percentages.

Given the limited sample size (*n* = 100) and the small number of events in several subgroups, statistical analyses were primarily descriptive and exploratory in nature.

Associations between categorical variables were assessed using the chi-square (χ^2^) test of independence, and effect size was quantified using Cramer’s V coefficient. Chi-square analyses were applied for exploratory purposes only, and all results were interpreted with caution in light of small subgroup sizes. Log-linear models were applied to explore multilateral interactions between selected categorical variables. Heatmaps were generated to visualize distributions and association patterns across subgroups. No multivariable predictive modeling (e.g., logistic regression) was performed, as the limited number of complication events and the fragmentation of predictors did not allow for robust multivariate analysis.

Descriptive and inferential statistical analyses were performed in Microsoft Excel, while graphical visualizations (heatmaps) were created in Python (Matplotlib). Statistical significance was set at *p* < 0.05, with emphasis placed on observed patterns rather than inferential generalization.

### 2.5. Ethical Approval

The study complied with the principles of the Declaration of Helsinki. Because the study used anonymized retrospective data, individual informed consent was not required. The research protocol was approved by the Institutional Ethics Committee (No. 12869/6 November 2023).

## 3. Results

### 3.1. Sociodemographic Characteristics

**Figure 1:**
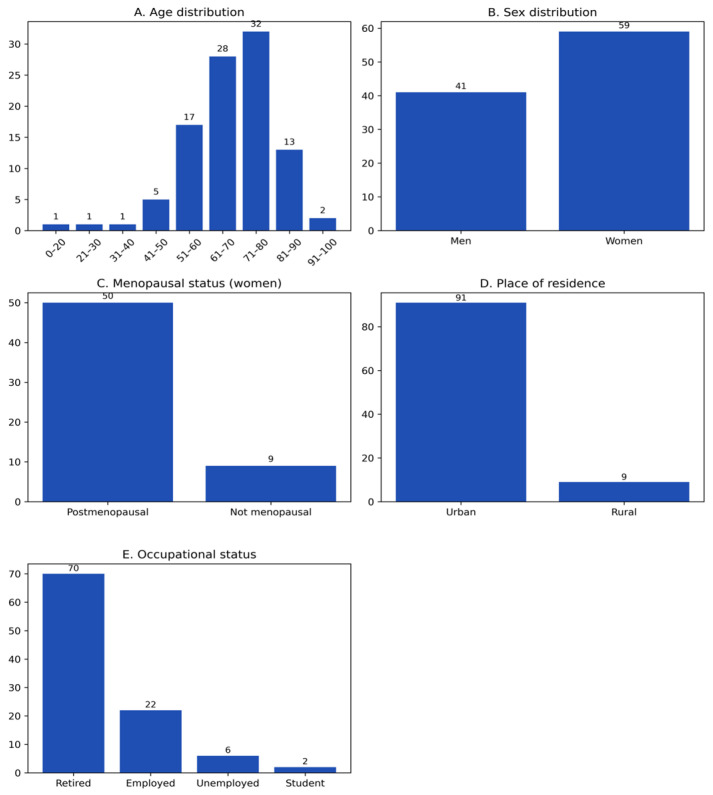
Demographic Profile of the Herpes Zoster Cohort: Age, Sex, Menopause Status, Residence, and Occupational Categories (Panels (**A**–**E**)).

The study cohort included 100 patients diagnosed with HZ, aged between 11 and 94 years. The mean age was 69.2 years, and the median age was 68 years, indicating a distribution strongly skewed toward older age groups. The age-group distribution was markedly heterogeneous, showing a pronounced concentration of cases in older age categories (Figure 1A). The highest proportion of cases occurred in the 71–80-year group (32%), followed by 61–70 years (28%) and 51–60 years (17%), whereas only three cases (3%) were identified below 40 years of age. Overall, more than three-quarters of patients (77%) were aged ≥60 years, highlighting a clear age-related clustering of HZ in this cohort.

Although the age distribution differed substantially from a uniform pattern (χ^2^ > 150; *p* < 0.0001), this analysis was considered descriptive and exploratory, reflecting the uneven age structure of the cohort rather than serving as an inferential comparison.

Sex distribution showed a predominance of females (59%) compared with males (41%) (Figure 1B), corresponding to an F:M ratio of approximately 1.44:1. The observed difference suggested a female predominance; however, the deviation from an equal distribution did not reach conventional statistical significance (χ^2^ (1) = 3.24; *p* ≈ 0.072; 95% CI for the proportion of females: 49.4–68.6%). Among the female patients (*n* = 59), 84.7% (*n* = 50) reported postmenopausal status, while 15.3% (*n* = 9) were premenopausal (Figure 1C). This finding reflects the older age profile of the cohort and the predominance of women in postmenopausal age groups, rather than implying an independent causal association.

Most patients resided in urban areas (91%), whereas only 9% lived in rural settings (Figure 1D). This marked imbalance suggests a higher representation of urban cases in this single-center cohort, potentially influenced by healthcare accessibility and referral patterns. The comparison with a theoretical equal distribution yielded a large χ^2^ value (χ^2^ = 67.24; *p* < 0.0001); however, this result should be interpreted descriptively, as it reflects population concentration rather than population-based incidence. Regarding occupational status (Figure 1E), retired individuals represented the majority (70%), followed by employed persons (22%), those without occupation (6%), and students/pupils (2%). This distribution mirrors the advanced age structure of the cohort and underscores the predominance of HZ among older, retired individuals.

The distribution was highly unbalanced (χ^2^ (3) = 95.68; *p* < 0.0001). This result is therefore interpreted as a descriptive characteristic of the cohort rather than as an inferential association.

### 3.2. Lifestyle and Behavioral Factors

**Figure 2:**
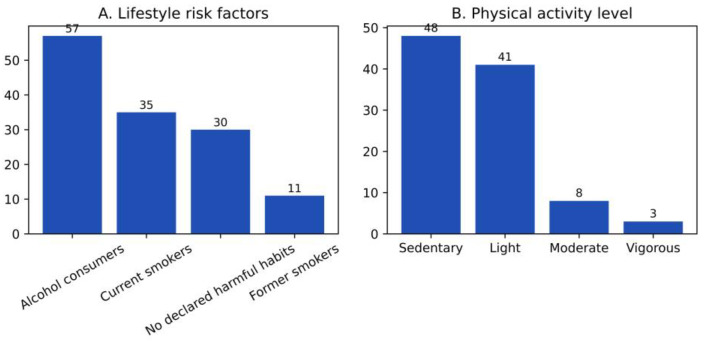
Distribution of Lifestyle Risk Factors and Physical Activity Categories in the Herpes Zoster Cohort (**A**,**B**).

The analysis of health-related behavioral factors revealed substantial heterogeneity in smoking status and alcohol consumption (Figure 2A). Overall, 57% of patients reported occasional alcohol use, 35% were active smokers, 30% reported no harmful habits, and 11% were former smokers. These frequencies illustrate a diverse distribution of lifestyle behaviors among patients diagnosed with HZ in this single-center cohort.

The observed distribution differed markedly from a uniform pattern (χ^2^ (3) = 41.52; *p* < 0.0001); however, this comparison is reported descriptively and reflects the uneven representation of lifestyle categories rather than an inferential assessment of behavioral risk.

Given the retrospective nature of data collection and the categorical classification of behaviors, these findings should be interpreted as prevalence patterns rather than causal associations.

Physical activity levels also showed an uneven distribution (Figure 2B). Most patients reported a sedentary lifestyle (48%) or light physical activity (41%), whereas only 8% engaged in moderate activity and 3% in vigorous activity. This pattern indicates a predominance of low physical activity levels within the cohort, consistent with its older age structure. The distribution across activity categories was highly unbalanced (χ^2^ (3) = 52.32; *p* < 0.0001), this result is presented as a descriptive characteristic of the cohort rather than as evidence of an inferential association between physical activity and HZ occurrence or severity.

The findings primarily highlight lifestyle patterns observed in this population and should be considered hypothesis-generating.

### 3.3. Anthropometric Parameters

**Figure 3:**
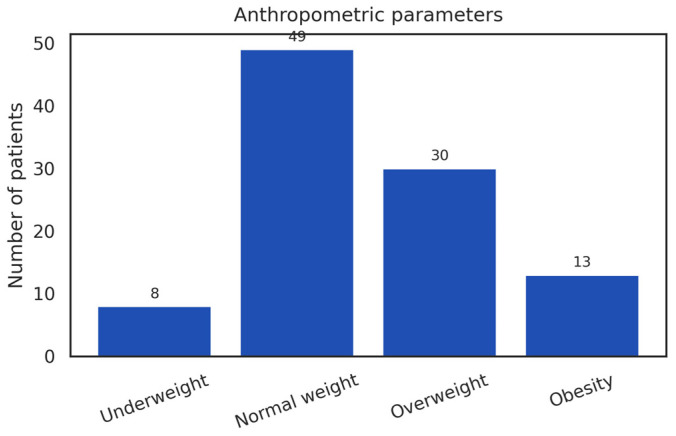
Anthropometric Profile of Patients with Herpes Zoster.

In the analysis of body weight status, calculated according to the World Health Organization and CDC recommendations using the standard BMI classification, 49% of patients were normal weight, 30% overweight, 13% obese, and 8% underweight (Figure 3). Overall, 43% of the cohort exhibited excess body weight, defined as overweight or obesity, reflecting the metabolic profile of this patient population.

The distribution across BMI categories was uneven, with a predominance of normal-weight and overweight individuals, while obesity and underweight were less frequently observed. The distribution differed markedly from a uniform pattern (χ^2^ (3) = 41.36; *p* < 0.0001), this comparison is presented descriptively and reflects the underlying structure of the cohort rather than an inferential association between BMI category and herpes zoster occurrence or severity.

Given the retrospective design and the use of BMI as a categorical anthropometric marker, these findings should be interpreted as descriptive prevalence patterns and considered hypothesis-generating rather than indicative of causal or predictive relationships.

### 3.4. Clinical Presentation

**Figure 4:**
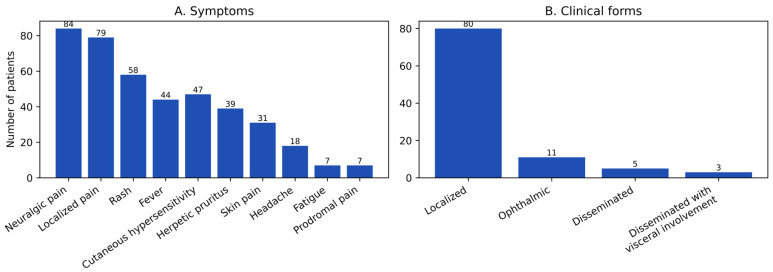
Clinical Spectrum of Herpes Zoster: Symptoms (**A**) and Clinical Forms (**B**).

The analysis of the cohort revealed a clinical profile dominated by the characteristic cutaneous and pain-related manifestations of HZ (Figure 4A). At admission, 84% of patients presented with vesicular eruptions, and 79% reported localized pain of varying intensity (95% CI: 76.8–91.2% and 71.0–87.0%, respectively). These findings illustrate the typical clinical presentation of HZ in this cohort, consistent with established diagnostic patterns. Constitutional symptoms were moderately frequent: 58% reported asthenia and 47% were febrile (95% CI: 48.3–67.7% and 37.2–56.8%). Local inflammatory signs included erythema (43%; 95% CI: 33.3–52.7%), hyperesthesia (39%; 95% CI: 29.4–48.6%), and pruritus (31%; 95% CI: 21.9–40.1%). Non-specific manifestations—headache, localized edema, and photophobia—were less common, with prevalence rates between 7 and 10% (approximate 95% CI: 2.0–15.9%). The observed symptom distribution corresponds to the expected spectrum of HZ-related manifestations at hospital admission and is reported descriptively, without implying differential severity or prognostic stratification.

Regarding the clinical forms of disease (Figure 4B), the majority of cases were classified as localized HZ (81%). Less frequent presentations included ophthalmic HZ (11%), disseminated HZ (5%), and disseminated HZ with varicelliform eruption (3%), outlining the clinical heterogeneity within the cohort.

The distribution of clinical forms was markedly uneven (χ^2^ (3) = 168.64; *p* < 0.0001). This comparison is presented descriptively and reflects the predominance of localized HZ within this hospital-based cohort rather than serving as an inferential assessment of disease severity or risk.

### 3.5. Disease Evolution and Complications

**Figure 5:**
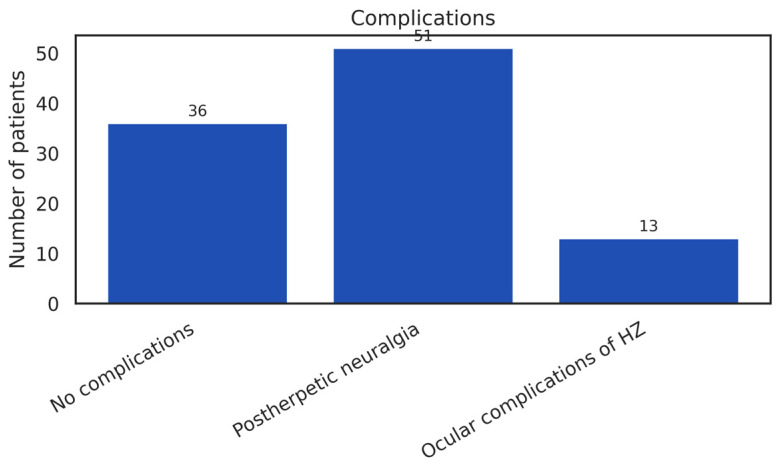
Frequency of Herpes Zoster Complication Types.

Patients were classified into three categories of clinical evolution: no recorded complications (67%), postherpetic neuralgia (PHN) (23%), and ocular complications (10%) (Figure 5). These proportions describe the distribution of clinical outcomes observed within the cohort.

PHN was more frequently observed among older patients, whereas no complications were recorded in patients under 40 years of age. This age-related pattern is reported descriptively and reflects the age structure of the cohort rather than implying an inferential association between age and disease evolution.

### 3.6. Epidemiological Characteristics

**Figure 6:**
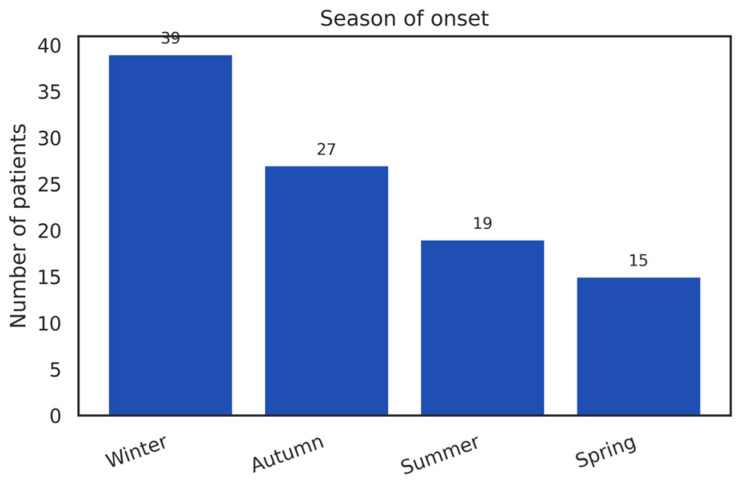
Distribution of Herpes Zoster by Season of Onset.

In this cohort, the seasonal distribution of HZ cases was uneven (Figure 6). A higher proportion of diagnoses occurred during winter (39%), predominantly in January–February, followed by autumn (27%), summer (19%), and spring (15%). These frequencies describe the temporal distribution of cases observed within this single-center cohort.

The comparison with a theoretical uniform seasonal distribution yielded a χ^2^ value of 13.44 (*p* ≈ 0.0038); however, this result is reported descriptively and reflects the uneven temporal clustering of cases rather than an inferential association between seasonality and HZ risk.

Accordingly, the observed seasonal pattern should be interpreted as a descriptive epidemiological characteristic of the cohort and considered hypothesis-generating.

### 3.7. Associations Between Demographic, Lifestyle, Anthropometric, Behavioral, and Seasonal Factors and Clinical Complications of Herpes Zoster

#### 3.7.1. Association Between Age Group and Herpes Zoster Complications

**Figure 7:**
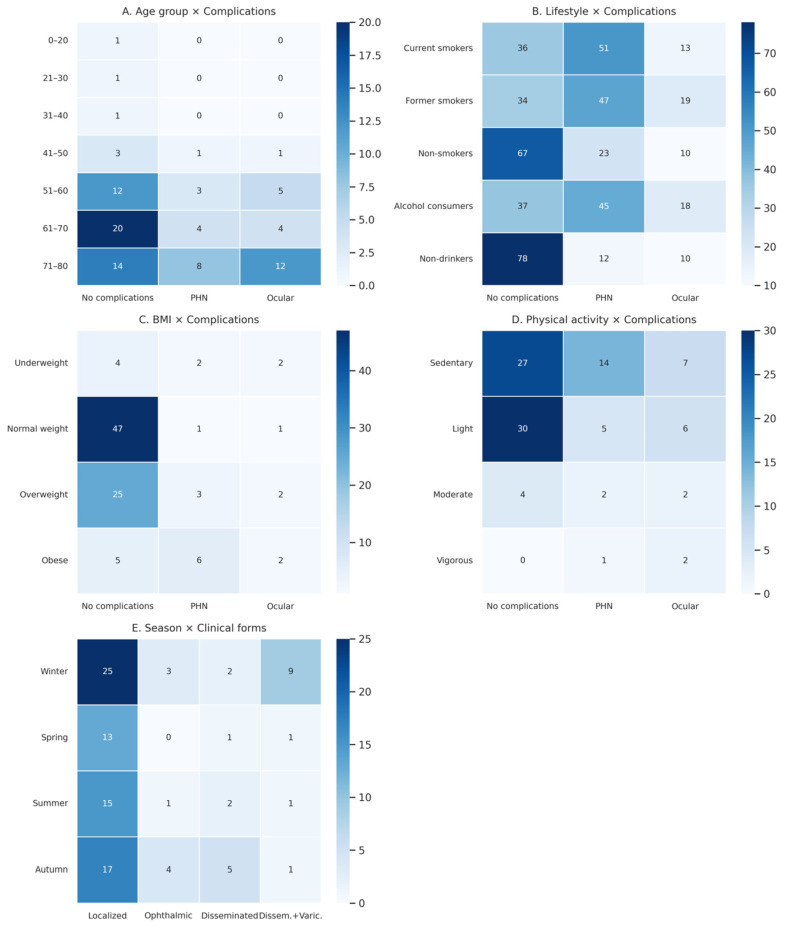
Heatmap analysis of key risk factors and clinical outcomes in the herpes zoster cohort. (**A**) Age group × complications; (**B**) lifestyle factors × complications; (**C**) BMI × complications; (**D**) physical activity × complications; (**E**) season × clinical forms.

The distribution of cases across age intervals and complication types is shown in Figure 7A. In younger age groups (0–40 years), only uncomplicated courses were reported. The first complicated cases appeared in the 41–50-year group, including one case of postherpetic neuralgia (PHN) and one ocular complication. The largest absolute number of cases, including both uncomplicated and complicated forms, was observed between 51 and 80 years of age, with the highest number of uncomplicated cases in the 61–70-year group (20 cases). The highest counts of PHN (8 cases) and ocular involvement (12 cases) were observed in the 71–80-year group, reflecting the older age structure of the cohort.

The chi-square test did not identify a statistically significant association between categorized age groups and clinical evolution (χ^2^ (12) = 8.53; *p* = 0.743), and Cramer’s V (0.22) indicated a weak association.

While the descriptive distribution suggests a higher frequency of complications at older ages, the limited size of several age-specific subgroups restricts the ability to demonstrate a robust statistical relationship. These findings are therefore reported descriptively and should be interpreted as exploratory.

#### 3.7.2. Association Between Lifestyle Factors and Herpes Zoster Complications

##### Smoking Status

The relationship between smoking status and clinical evolution is shown in Figure 7B. Among active smokers, PHN was the dominant complication (51%), followed by uncomplicated forms (36%) and ocular complications (13%). Former smokers displayed a similar profile, with elevated rates of PHN (47%) and the highest proportion of ocular involvement (19%). Nonsmokers exhibited the most favorable evolution: 67% uncomplicated, with markedly lower frequencies of PHN (23%) and ocular complications (10%).

The chi-square analysis indicated a statistically significant difference in the distribution of complication types across smoking categories (χ^2^ (4) = 29.36; *p* = 6.6 × 10^−6^), with Cramer’s V = 0.22, suggesting a weak-to-moderate association.

Given the retrospective design, small subgroup sizes, and categorical classification of smoking status, this finding should be interpreted cautiously and considered descriptive rather than indicative of a causal relationship.

##### Alcohol Consumption

Alcohol consumption was associated with increased complication rates: 45% PHN and 18% ocular involvement, compared to only 37% uncomplicated evolution. In contrast, non-consumers had 78% uncomplicated evolution, with substantially lower PHN (12%) and ocular complications (10%).

The chi-square test showed a significant difference in the distribution of complication types across alcohol consumption categories (χ^2^ (2) = 36.01; *p* = 1.5 × 10^−8^), with Cramer’s V = 0.42, indicating a moderate association.

These results reflect observed differences in clinical evolution across exposure categories but should be interpreted as exploratory patterns rather than evidence of causality.

##### Combined Model (Smoking and Alcohol)

An exploratory combined analysis of smoking and alcohol consumption (5 × 3 design) demonstrated a non-uniform distribution of clinical evolution across lifestyle categories (χ^2^ (8) = 71,78; *p* = 2.17 × 10^−12^), with Cramer’s V = 0.27.

This combined analysis highlights descriptive clustering patterns within the cohort; however, given the limited number of complication events and fragmented subgroups, the results are presented for exploratory purposes only and do not support multivariable or predictive inference.

#### 3.7.3. Association Between Weight Status and Herpes Zoster Complications

The distribution of cases is presented in Figure 7C. Normal-weight patients showed the highest proportion of uncomplicated clinical courses (47%), whereas overweight and obese patients exhibited higher proportions of PHN. Underweight individuals demonstrated a relatively uniform distribution across all three evolution categories. The chi-square analysis indicated a non-uniform distribution of clinical evolution across BMI categories (χ^2^ (6) = 18.92; *p* = 0.004), with Cramer’s V = 0.24, indicating a weak-to-moderate association.

Given the limited sample size and the categorical use of BMI as a surrogate anthropometric marker, this finding is reported descriptively and should be interpreted as an exploratory pattern rather than evidence of a causal or predictive relationship between weight status and HZ complications.

#### 3.7.4. Association Between Physical Activity Level and Herpes Zoster Complications

The distribution of clinical outcomes according to physical activity level is shown in Figure 7D. Sedentary patients exhibited the highest frequency of PHN (29.1%) and a substantial proportion of ocular complications (14.5%). Light physical activity was associated with the most favorable evolution (73.1% uncomplicated). Moderate physical activity showed a more balanced distribution of outcomes. Vigorous physical activity was reported in only three patients, among whom ocular complications predominated. The chi-square test indicated a statistically significant difference in the distribution of clinical evolution across physical activity categories (χ^2^ (6) = 15.82; *p* = 0.014), with Cramer’s V = 0.28, indicating a moderate association. The very small size of some subgroups, particularly the vigorous activity category, substantially limits inferential interpretation, and the findings are therefore presented as descriptive and hypothesis-generating rather than as evidence of a robust association.

#### 3.7.5. Seasonal Distribution of Clinical Forms of Herpes Zoster

The seasonal distribution of clinical forms is shown in Figure 7E. Localized HZ predominated across all seasons, with the highest number of cases recorded in winter. Complicated forms, including ophthalmic and disseminated HZ, showed a modest tendency to cluster during the colder months; however, these patterns were not consistent across all seasons. The chi-square test did not identify a statistically significant association between season of onset and clinical form (χ^2^ (9) = 13.81; *p* = 0.129). Cramer’s V = 0.21 indicated a weak association. The observed seasonal variation in clinical forms is presented descriptively and should be interpreted cautiously, without implying a seasonal effect on disease severity.

## 4. Discussion

### 4.1. Impact of Age on the Incidence and Complications of Herpes Zoster

Figure 7A illustrates the distribution of age groups and complication types, highlighting a marked accumulation of cases within the 60–80-year intervals, encompassing both uncomplicated courses and major complications, including postherpetic neuralgia (PHN) and ocular involvement. In the age groups below 40 years, only uncomplicated clinical courses were observed, reflecting the age structure of the cohort.

The age distribution was highly uneven, with a pronounced concentration of cases in older age categories (χ^2^ > 150; *p* < 0.0001), This finding describes the demographic structure of the cohort and is consistent with the well-documented predominance of herpes zoster among older adults, rather than representing an inferential association. Large epidemiological studies indicate that more than half of herpes zoster cases occur in individuals over 60 years of age, with mean ages in most cohorts centered around the sixth decade of life [18]. Similarly, Hsiao et al. reported a marked increase in incidence after the age of 50, with individuals aged ≥50 years exhibiting substantially higher rates of disease compared with younger adults [19]. Comparable trends have been described by Kawai et al., who attributed this pattern to age-related decline in cell-mediated immunity [6].

From a biological perspective, these epidemiological patterns are commonly explained by immunosenescence. Oh et al. demonstrated that aging is associated with progressive deterioration of T-cell–mediated immune responses, reducing control over latent varicella-zoster virus and facilitating viral reactivation [13]. Levin et al. further documented a gradual decline in antiviral immune responses after the age of 60 [20]. The age distribution observed in our southeastern Romanian cohort is compatible with these established mechanisms.

Regarding disease evolution, the descriptive distribution suggested higher frequencies of PHN and ocular complications in the 60–80-year age groups. However, no statistically significant association was identified between categorized age and clinical evolution in chi-square analysis (*p* > 0.05), likely due to the limited number of cases within certain age–outcome subgroups. This absence of statistical significance should be interpreted in the context of sample size constraints and does not negate the descriptive trends observed.

Extensive evidence from larger cohorts supports an age-related increase in disease severity. John and Canaday demonstrated that immunosenescence affects both viral reactivation and the extent of neural injury underlying PHN [21], while Forbes et al. reported a sharp rise in PHN incidence after the age of 60 [22]. Kong et al. identified a peak prevalence of ophthalmic herpes zoster in individuals aged 60–70 years, particularly among those with comorbidities [23]. These findings are consistent with the age-related distribution of complications observed in our cohort, where most ocular complications occurred in patients aged 71–80 years.

Given the limited statistical power, interpretation of age-related patterns must account for sample size constraints. Large-scale population studies, such as that conducted by Jung et al. [24], have consistently shown that uncomplicated herpes zoster predominates at younger ages, whereas the frequency of severe complications increases exponentially after the age of 60, a pattern replicated across both European and Asian cohorts.

### 4.2. Influence of Sex and Menopausal Status on Susceptibility to Herpes Zoster

Figure 7B illustrates the distribution of cases according to sex and menopausal status, revealing the predominance of HZ among women and the high proportion of postmenopausal female patients. In our cohort, women were more frequently affected than men—a trend at the margin of statistical significance but fully consistent with international epidemiologic data. Large population-based studies have consistently reported a slightly higher risk of herpes zoster in women compared with men, with an increase of approximately 20–30% after adjustment for age and other relevant risk factors [6,25,26]. This sex-related difference has been widely observed across diverse populations, although its underlying determinants remain incompletely defined.

Among the women included in this study, 84.7% were postmenopausal, a proportion that reflects the older age distribution of the cohort. Rather than indicating menopause as an independent risk factor, this observation suggests a contextual association between menopausal status and HZ occurrence, likely mediated by age-related biological changes. Similar age- and sex-specific patterns have been reported in previous studies, including the retrospective analysis by Ozturk et al., which identified a higher frequency of HZ in women aged 46–55 years, corresponding to the menopausal transition, and proposed that hormonal fluctuations during this period may influence viral reactivation [26,27].

From a mechanistic perspective, estrogens exert well-documented immunomodulatory effects on both innate and adaptive immunity, including the regulation of T-cell proliferation, maintenance of memory T-cell populations, and modulation of antiviral cytokine production. Experimental studies indicate that estrogen signaling enhances IL-2 receptor expression and supports IFN-γ–mediated antiviral immune responses [28,29,30]. Accordingly, the decline in estrogen levels observed during menopause may contribute to alterations in immune competence that overlap with age-related immunosenescence, without implying a direct causal relationship.

Postmenopausal estrogen decline has also been associated with a pro-inflammatory milieu characterized by increased IL-6, TNF-α, and CRP levels, a phenomenon often described as “inflammaging” [31]. This immunologic environment may facilitate viral reactivation and influence clinical expression while remaining part of a broader age-related immune remodeling process. In addition, estrogen deficiency has been linked to changes in peripheral nerve integrity and neuroinflammatory responses, which may contribute to the development of postherpetic neuralgia (PHN), as suggested by previous clinical observations [14,21].

Taken together, these findings support the interpretation that female sex and menopausal status are associated with herpes zoster occurrence within a broader framework of immunosenescence and age-related immune modulation. Menopause should therefore be viewed as a biologically plausible contextual factor rather than a confirmed independent determinant of disease risk. These considerations highlight the importance of individualized risk assessment in women, integrating age, immune status, comorbidities, and hormonal milieu, particularly in regions with a high burden of HZ such as southeastern Romania.

It should also be acknowledged that the higher frequency of herpes zoster reported in women may be partially influenced by non-biological factors. Women are generally more likely to seek medical care and to present earlier for dermatological symptoms, which may increase diagnostic recognition, particularly in milder or atypical cases. Consequently, differences in healthcare-seeking behavior, access to medical services, and diagnostic practices may contribute to the observed female predominance, alongside biological factors such as hormonal and immune differences.

### 4.3. Distribution of Cases in Urban Areas and Implications for Epidemiological Reporting

Figure 1D illustrates the marked contrast between urban and rural settings, highlighting the overwhelming predominance of cases originating from urban areas. This distribution describes the residential profile of patients included in the study and reflects a pronounced imbalance in case origin.

The comparison with a theoretical uniform distribution yielded a large χ^2^ value (χ^2^ = 67.24; *p* < 0.0001). This result is presented descriptively and reflects the structure of the cohort rather than an inferential estimate of population-level incidence.

A plausible explanation for the observed urban predominance relates to differences in healthcare accessibility and diagnostic pathways. Urban settings typically provide greater availability of medical services and specialist care, which may facilitate earlier clinical presentation and diagnostic confirmation of herpes zoster. Higher levels of health literacy and more frequent healthcare utilization may further contribute to increased case detection in urban populations. Similar patterns have been reported for other clinically confirmed infectious diseases, in which diagnoses are more frequently recorded in settings with better healthcare infrastructure [32].

Comparable urban–rural disparities have been documented across diverse geographic regions, suggesting that such differences often reflect variations in surveillance performance and healthcare access rather than true differences in disease incidence [33]. The observed distribution in this cohort should be interpreted cautiously and primarily as an indicator of case ascertainment patterns.

At the same time, the possibility of genuine epidemiological differences between urban and rural settings cannot be excluded. Socioeconomic conditions, cumulative stress exposure, higher comorbidity burden, and environmental factors—more frequently encountered in urban environments—have been proposed as contextual contributors to viral reactivation. Conversely, rural settings may face structural barriers to healthcare access, where delayed care-seeking, self-management, and underdiagnosis can lead to substantial underreporting, a phenomenon well documented in surveillance systems for clinically diagnosed infectious diseases [34].

These observations suggest that place of residence may affect case identification through differences in healthcare access and reporting practices, and should be interpreted cautiously within the context of a small, single-center cohort.

### 4.4. Role of Lifestyle Behaviors in the Severity of HZ

The analysis of lifestyle behaviors in the present cohort revealed a heterogeneous distribution of factors known to modulate immune function, with a predominance of individuals reporting alcohol consumption and active or former smoking (Figure 2A). Interpretation of these findings requires careful integration with existing epidemiological evidence, which remains inconsistent regarding the relationship between lifestyle factors and herpes zoster incidence.

Meta-analytic evidence from Marra et al. indicates that active smoking is not associated with an increased risk of HZ, although a modest elevation in risk has been observed among former smokers, potentially reflecting the cumulative effects of prior tobacco exposure [35]. Other cohorts identified a predominance of nonsmokers among HZ patients, without evidence of a clear link between smoking and VZV reactivation. In Taiwan, Dai et al. [36] even reported an inverse association, with active smokers exhibiting a lower probability of HZ, a finding replicated in the Shozu Herpes Zoster Study in Japan [37]. Regarding alcohol consumption, most analyses suggest that only chronic or excessive alcohol exposure has meaningful immunological consequences, whereas occasional consumption has a far less clear impact [38].

In contrast to the heterogeneous evidence regarding disease incidence, the present cohort displayed descriptive patterns suggesting differences in clinical evolution across lifestyle categories. According to the heatmap (Figure 7B), active smokers, former smokers, and alcohol consumers displayed a higher frequency of complicated outcomes, particularly PHN and ocular involvement, compared with nonsmokers and abstainers, who showed a predominance of uncomplicated forms. These observations are reported descriptively and should be interpreted as exploratory, given the retrospective design and limited subgroup sizes.

From a biological standpoint, a dissociation between disease incidence and disease severity is plausible. Chronic smoking is associated with sustained oxidative stress, depletion of antioxidant systems (notably reduced glutathione), and activation of NF-κB–dependent pro-inflammatory pathways, resulting in a state of chronic low-grade inflammation. This immunological milieu is characterized by elevated circulating IL-6, TNF-α, and CRP levels, together with qualitative alterations in T-cell populations, including reduced naïve T-cell pools, expansion of exhausted memory clones, and diminished IFN-γ and IL-2 production—key mechanisms involved in the control of latent viral infections [22,39]. Cigarette smoke also impairs dendritic cell and NK cell function, reducing immune surveillance and the capacity to maintain VZV latency [39]. At the vascular level, nicotine and tobacco combustion products induce endothelial dysfunction, microangiopathy, and peripheral hypoperfusion, mechanisms that can aggravate neuroinflammation and contribute to the persistence of neuropathic pain characteristic of PHN [22,40].

Chronic alcohol consumption exerts convergent effects on immune surveillance. Alcohol disrupts intestinal barrier integrity and promotes bacterial translocation, leading to endotoxemia and chronic immune activation followed by functional exhaustion of innate immune cells [41,42]. It also interferes with T- and B-cell differentiation and antiviral cytokine production, impairing host control of latent viral infections and delaying immune clearance during reactivation [42]. At the neurological and vascular levels, chronic alcohol exposure is associated with peripheral neuropathy, microangiopathy, and nutritional deficiencies—particularly B-vitamin depletion—which may exacerbate PHN severity and increase the likelihood of complications [41,43].

Within this framework, the higher proportion of uncomplicated outcomes observed among nonsmokers and individuals without harmful behaviors in the present cohort is compatible with contemporary pathogenic models of herpes zoster, in which immunological dysfunction—driven by immunosenescence, systemic inflammation, and chronic stress—represents a central vulnerability, while lifestyle behaviors act as potential modifiers rather than primary determinants [21,39,40,41,42,43].

Physical activity was examined in light of its recognized bidirectional effects on immune function. In this cohort, physical activity levels were markedly unbalanced, with a predominance of sedentary individuals (48%), followed by light (41%), moderate (8%), and vigorous activity (3%) (Figure 2B). This distribution reflects the age structure of the cohort and was reported descriptively (χ^2^ (3) = 52.32; *p* < 0.0001).

Exploratory analysis of clinical evolution by physical activity level (Figure 7D) revealed distinct descriptive patterns. Light physical activity was associated with the highest proportion of uncomplicated cases, whereas sedentary individuals exhibited higher frequencies of PHN and ocular complications. Vigorous physical activity was rare and occurred in only a few patients, among whom complicated outcomes predominated. Given the very small size of this subgroup, these findings should be interpreted cautiously and considered hypothesis-generating.

Moderate physical activity is widely recognized as beneficial for antiviral immunity, enhancing natural killer cell mobilization, T-cell trafficking, and lymphatic circulation while reducing systemic low-grade inflammation [44,45]. These mechanisms are consistent with the more favorable clinical profiles observed among individuals engaging in light activity. Conversely, intense or sustained exercise may induce transient immunosuppression—the so-called “open window” phenomenon—characterized by elevated cortisol and IL-6 levels and reduced lymphocyte and NK-cell activity, potentially facilitating herpesvirus reactivation [46,47].

Sedentarism, on the other hand, promotes inflammaging, metabolic dysregulation, and progressive decline in immune cell competence, particularly within T- and NK-cell compartments [48]. This inflammatory profile may impair control of latent VZV and amplify susceptibility to neuropathic complications such as PHN [21].

Taken together, the descriptive patterns observed in this cohort suggest that both extremes of physical activity—sedentarism and vigorous exercise—may be associated with less favorable clinical evolution, whereas light-to-moderate activity is associated with more benign outcomes. These observations are consistent with immunological evidence and international literature while remaining exploratory and constrained by the limitations inherent to a small, single-center, retrospective study.

### 4.5. Implications of Weight Status and Metabolic Dysfunction in HZ Complications

The relationship between weight status and the risk of HZ remains complex and incompletely elucidated, despite an increasing number of population-based and immunometabolic studies. Most large epidemiological analyses suggest that body mass index (BMI) is not a major determinant of HZ onset. For example, in the Danish cohort reported by Sigrun [38] the distribution of normal-weight, overweight, and obese individuals among HZ patients closely mirrored that of the general population, and multivariable adjustments did not alter this conclusion. However, more recent evidence from Chen et al. (2024) [49] suggests that obesity—particularly severe obesity—may be associated with higher herpes zoster incidence, indicating that the contribution of BMI to varicella-zoster virus (VZV) reactivation may vary across populations and metabolic contexts.

Additional studies provide more nuanced perspectives. In the Shozu Herpes Zoster Study, Kawahira et al. [41] observed a lower incidence of HZ among overweight women, suggesting a potential protective effect of moderate excess weight, possibly related to hormonal or cytokine-related mechanisms. This observation has not been consistently replicated in other cohorts, suggesting that the relationship between BMI and herpes zoster incidence may depend on population-specific characteristics and study design [41].

From an immunometabolic perspective, these descriptive patterns are compatible with emerging evidence—including Mendelian randomization analyses—indicating that excess adiposity, particularly in more severe forms, is associated with chronic low-grade inflammation, oxidative stress, impaired T-cell–mediated immunity, and alterations of the neuronal microenvironment. Rather than triggering varicella-zoster virus reactivation, these mechanisms are more likely to reduce the efficiency of cell-mediated antiviral surveillance and to modulate the severity of postherpetic complications, including PHN [15,22,49].

Within this context, the weight profile of our cohort, dominated by normal-weight and overweight individuals, with low prevalence of severe obesity or underweight, aligns with distributions reported in international studies (Figure 3). This pattern does not indicate an anthropometric profile specific to HZ patients in this region and supports the idea that BMI alone is not a primary determinant of viral reactivation.

When clinical evolution is considered (Figure 7C, heatmap), descriptive patterns suggest differences in disease severity across BMI categories. Normal-weight individuals predominantly experienced uncomplicated evolution, whereas overweight and obese patients exhibited higher proportions of PHN and, occasionally, disseminated forms. These observations are reported descriptively and should be interpreted cautiously, given the limited sample size and categorical use of BMI as a surrogate marker of metabolic status.

Within this framework, the findings suggest that body mass index does not drive herpes zoster occurrence but may influence clinical evolution by reflecting underlying metabolic dysfunction. Excess weight may act as a contextual modifier of neuronal and immunologic vulnerability, thereby increasing susceptibility to complications such as postherpetic neuralgia without serving as a direct trigger for varicella-zoster virus reactivation. These observations highlight the relevance of metabolic assessment in the clinical management of herpes zoster and point to the need for future studies integrating comprehensive inflammatory and metabolic biomarkers (e.g., IL-6, TNF-α, adipokines, TyG index, insulin resistance) to clarify the immunometabolic pathways involved in disease severity.

### 4.6. Distribution of Patients According to Frequency of Clinical Signs and Symptoms

The distribution of clinical signs and of symptoms in our cohort closely reflects the typical clinical spectrum of herpes zoster, characterized predominantly by cutaneous manifestations and neuropathic pain. As illustrated in Figure 5, vesicular eruption (84%) and localized pain (79%) were the most frequently recorded findings, describing the predominant symptom profile at presentation.

Neuropathic pain and cutaneous lesions are widely recognized as the dominant manifestations at disease onset, although their intensity and temporal relationship may vary substantially between individuals. In the present cohort, hyperesthesia was documented in 39% of patients, a proportion that falls within the range reported across previous observational studies.

Prodromal and constitutional symptoms, including asthenia (58%), fever (44%), and headache (31%), were also commonly recorded, reflecting the heterogeneity of early clinical manifestations in herpes zoster. Such variability in prodromal presentation has been consistently described in epidemiological and clinical studies, emphasizing that systemic symptoms may precede or accompany the onset of typical dermatomal eruptions.

Additional local inflammatory signs—erythema (47%), pruritus (39%), edema (7%), and photophobia (7%)—further illustrate the broad inflammatory spectrum associated with acute varicella-zoster virus reactivation. This symptomatic profile aligns with international reports describing a wide range of manifestations, from nonspecific prodromes to intense neuropathic pain and characteristic vesicular eruptions distributed along affected dermatomes [21].

Regarding clinical forms, the distribution observed in this cohort follows patterns commonly reported in epidemiological studies. Localized herpes zoster represented the predominant presentation, while ophthalmic herpes zoster (HZO) accounted for a moderate proportion of cases, and disseminated or varicelliform forms were infrequent. The proportion of HZO observed in this cohort (approximately 11%) lies within the broad range reported globally (2.5–20%) and is comparable to estimates from large multicenter analyses, including the study by Kong et al. (2020), which reported a prevalence of 7.9% with variation according to age and calendar period [23].

Disseminated and varicelliform presentations (approximately 5% and 3%, respectively) were uncommon, in agreement with international epidemiological data indicating that such forms occur predominantly in immunosuppressed contexts, although isolated cases in individuals without overt immunosuppression have also been documented [50,51].

### 4.7. Seasonal Patterns of Clinical Forms of HZ and Potential Explanatory Factors

The seasonal distribution observed in our cohort shows a pronounced peak during the cold season (39 cases in winter), followed by a decline in autumn (27 cases), summer (19 cases), and spring (15 cases) (Figure 6). This distribution is presented as a descriptive characteristic of the cohort and reflects temporal clustering of diagnosed cases rather than a population-level estimate of seasonal incidence. The observed pattern suggests that VZV reactivation is not uniform throughout the year and may reflect region-specific climatic conditions, healthcare-seeking behavior, and seasonal population dynamics.

Seasonal variation in herpes zoster incidence has been reported in previous studies, although the literature remains heterogeneous and often inconsistent across geographic regions. A Korean analysis of 1105 patients [24] revealed an almost uniform annual distribution with slight spring–summer predominance, while studies from Japan, Australia, and Taiwan reported increased summer incidence, possibly influenced by ultraviolet radiation exposure [52]. Other ecological analyses, such as that by Choi et al. (2019) [53], have associated increasing ambient temperature with higher HZ incidence, supporting the hypothesis that local microclimatic conditions may modulate seasonal patterns. In contrast, varicella infection exhibits far more consistent winter–spring seasonality, while herpes zoster demonstrates markedly variable temporal distributions. A nationwide Japanese study reported a modest but reproducible summer increase in HZ incidence [54], and time-series analyses by Lv et al. (2023) further demonstrated that temperature and meteorological variables significantly influence HZ incidence in a location-dependent manner [55].

The available evidence suggests that seasonality functions as a contextual and region-dependent modifier rather than as a universal determinant of herpes zoster occurrence.

Regarding disease severity, analysis of the heatmap (Figure 7E) did not identify significant differences between seasons. Localized HZ predominated across all seasons, HZO showed modest seasonal variation (slightly more frequent in winter and autumn), and disseminated or varicelliform cases were rare. Chi-square analysis did not demonstrate a statistically significant association between season and complication type, and effect size estimates indicated a weak association, supporting a primarily descriptive interpretation.

These observations are consistent with previous reports, including the study by Jung et al. [24] which described seasonal fluctuations in case numbers without parallel changes in disease severity, as well as multicenter analyses by Berlinberg et al. [52] where clinical presentation remained stable despite temporal variation in incidence.

In this cohort, the apparent clustering of disseminated or varicelliform cases during the cold season is noted as an exploratory observation; however, the very small number of such cases precludes any robust inferential conclusions. It is therefore more likely that this distribution reflects local characteristics of southeastern Romania—such as seasonal immune modulation (e.g., vitamin D status, intercurrent respiratory infections), environmental temperature, and healthcare utilization patterns—rather than a generalizable pathogenic mechanism.

Overall, the findings suggest that while the number of diagnosed herpes zoster cases varies seasonally in this hospital-based cohort, seasonality does not appear to substantially influence the severity or clinical form of disease. Intrinsic host-related factors, including age, immune status, and comorbidities, remain the dominant determinants of clinical evolution, with season acting as a secondary, modifying factor.

This study presents several limitations that should be taken into consideration. As a result, several analyses should be interpreted as descriptive and hypothesis-generating rather than as providing definitive inferential evidence. The relatively small sample size and the uneven distribution of certain subgroups (rare clinical forms, extreme physical activity levels, small weight-status categories) may limit the ability to detect subtle statistical differences. The retrospective nature of the study inherently limited the completeness and granularity of available clinical information. In particular, data on conditions known to impair cell-mediated immunity—including chronic viral infections, oncologic diseases, prior exposure to chemo- or radiotherapy, long-term systemic immunosuppressive treatments, biologic therapies, post-transplant status, and autoimmune disorders—were inconsistently documented across medical records and could not be reliably incorporated into the analysis. In addition, information regarding the timing of primary varicella infection, including infection occurring during infancy, was not routinely recorded and therefore could not be evaluated as a modifying factor in disease susceptibility or severity.

Additionally, the retrospective design did not allow for full control of other potential confounding factors, including cumulative exposure to smoking or alcohol, socioeconomic status, psychological stress, history of varicella infection in childhood, and vaccination status.

The predominantly urban distribution may also reflect differential access to medical services and healthcare-seeking behavior, as discussed above. Furthermore, the use of BMI as the sole metabolic marker does not capture the complexity of the inflammatory and immunometabolic mechanisms involved in herpes zoster progression.

Future research directions include expanding the analysis to larger and ideally multicenter cohorts to strengthen the external validity of the findings. Prospective studies are needed to allow for rigorous control of comorbidities and behavioral factors, as well as the integration of inflammatory and metabolic biomarkers (IL-6, TNF-α, CRP, adipokines, TyG index) to clarify the immunometabolic mechanisms that may influence HZ severity. Investigating the impact of vaccination and lifestyle interventions may further support the development of effective preventive strategies. Finally, the role of regional and sociodemographic factors deserves deeper investigation to understand how healthcare access shapes reporting and diagnosis patterns of this condition.

## 5. Conclusions

This study provides a comprehensive characterization of the clinical and epidemiological profile of herpes zoster in southeastern Romania and contributes meaningful insights to the current literature. Lifestyle factors exhibited a clear gradient of severity: smoking, alcohol consumption, and sedentary behavior were associated with higher rates of postherpetic neuralgia and ocular complications, whereas light physical activity correlated with the most favorable outcomes. The increased frequency of complications among individuals engaging in vigorous exercise is consistent with the well-established “open window” phenomenon described in exercise immunology.

From a metabolic perspective, overweight and obesity were linked to a greater burden of postherpetic neuralgia, suggesting that immunometabolic dysfunction may modulate disease severity. Demographic analyses revealed a predominance of urban cases and a strong concentration among older adults, supporting immunosenescence as a central driver of varicella-zoster virus reactivation. The notable proportion of postmenopausal women further reinforces the hypothesis that estrogen decline may increase susceptibility in females.

These findings enhance current understanding of the clinical, demographic, behavioral, and metabolic determinants of herpes zoster in an understudied Eastern European population. The results may provide a foundation for developing targeted prevention strategies, improving patient education, and implementing risk-stratification approaches in clinical practice.

## Data Availability

Data are not publicly available due to privacy and ethical restrictions.

## Abbreviations

The following abbreviations are used in this manuscript:

HZ	Herpes zoster
VZV	varicella–zoster virus
PHN	postherpetic neuralgia
HZO	ophthalmic Herpes zoster
